# Supplementary figures and images for: NAB2-STAT6 drives an EGR1-dependent neuroendocrine program in solitary fibrous tumors
Source: eLife. 2025 Aug 28;13:RP98072. doi: 10.7554/eLife.98072 (PMC12393881; doi:10.7554/eLife.98072)

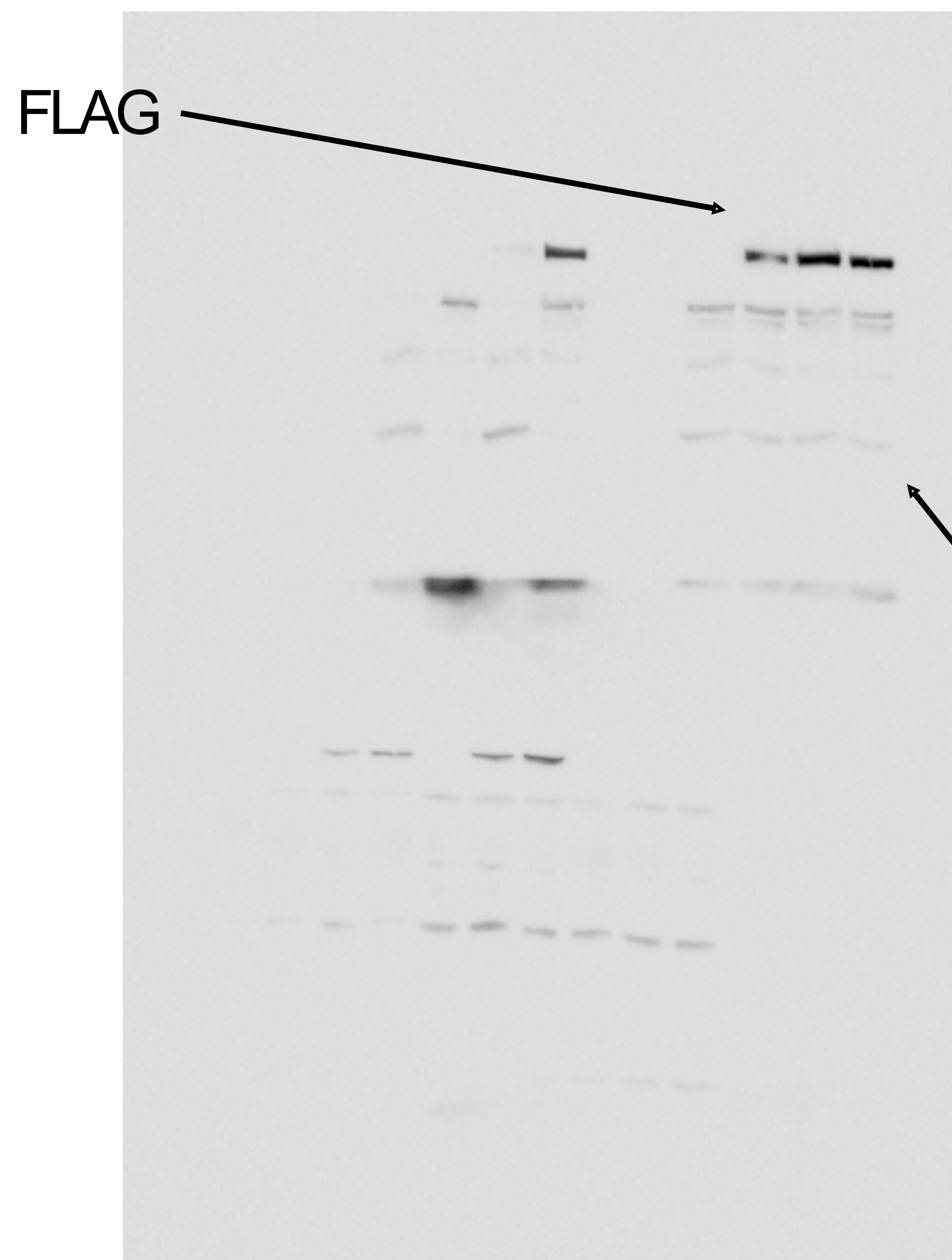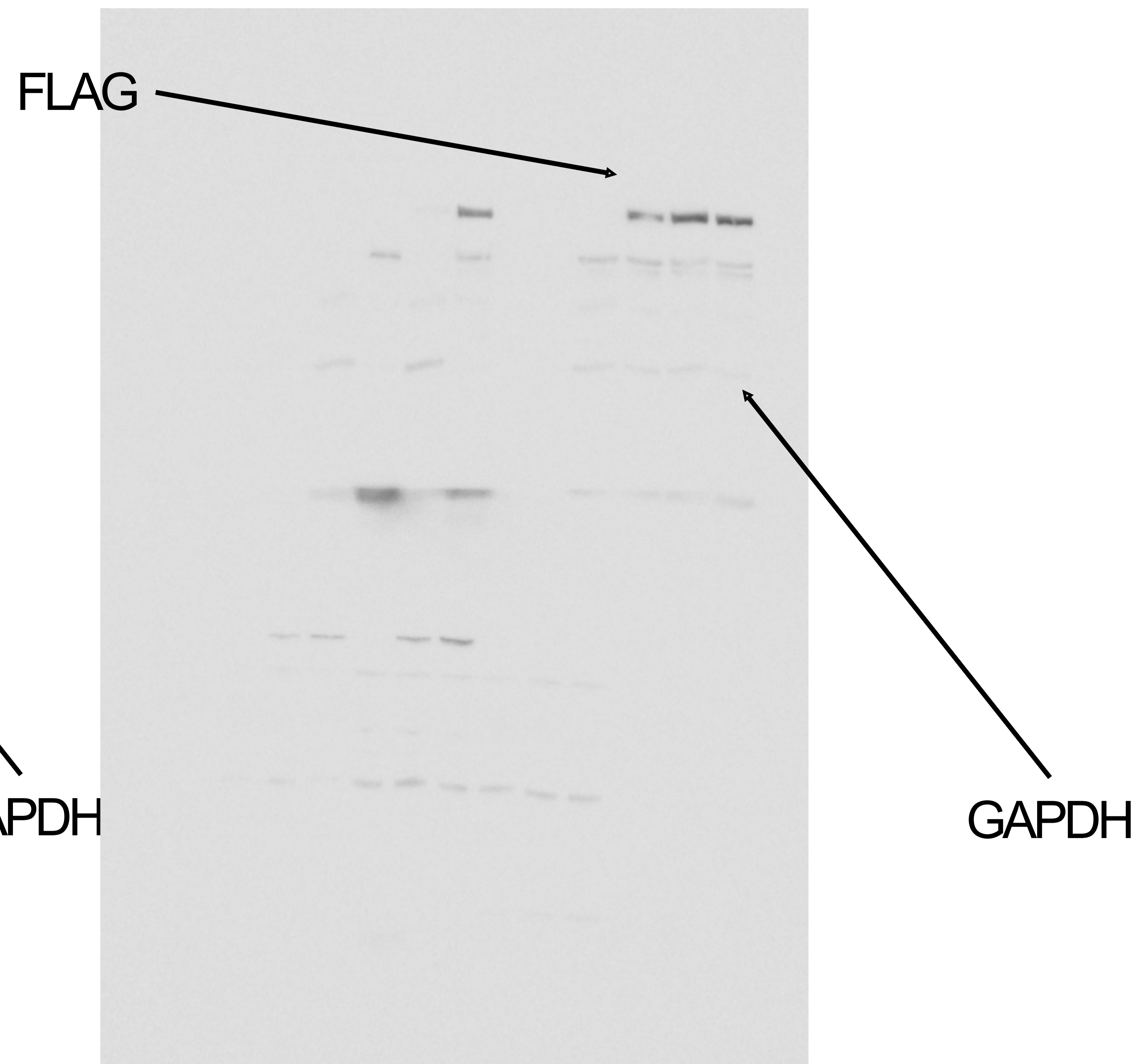

Figure 2, Source data 1. Original membranes corresponding to figure 2, panel A.

Supplement: Figure 2—source data 1. [file elife-98072-fig2-data1.zip › Figure2_sourcedata1.pdf]

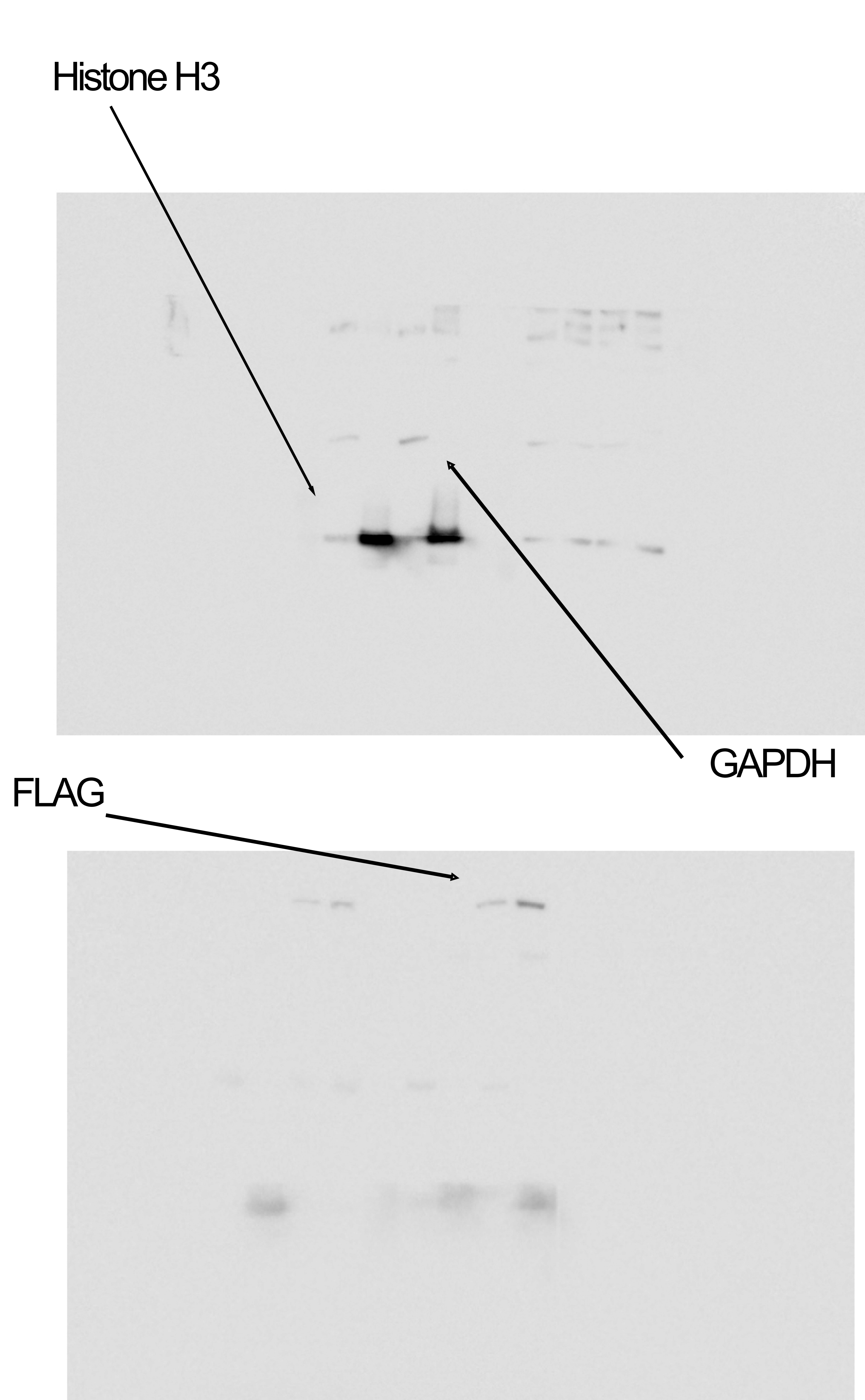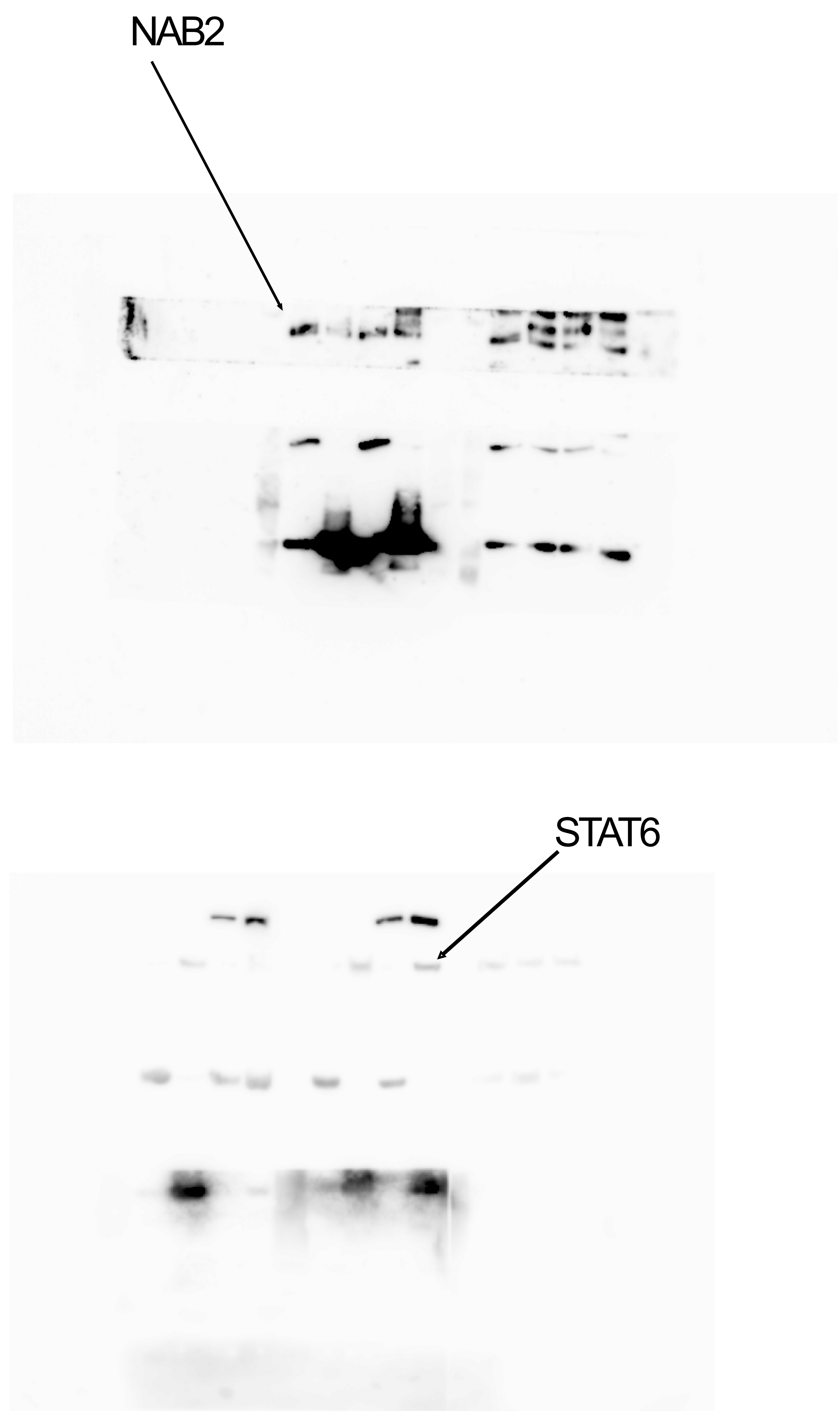

Figure 5, Source data 1. Original membranes corresponding to figure 5 , panel B.

Supplement: Figure 5—source data 1. [file elife-98072-fig5-data1.zip › Figure5_sourcedata1.pdf]

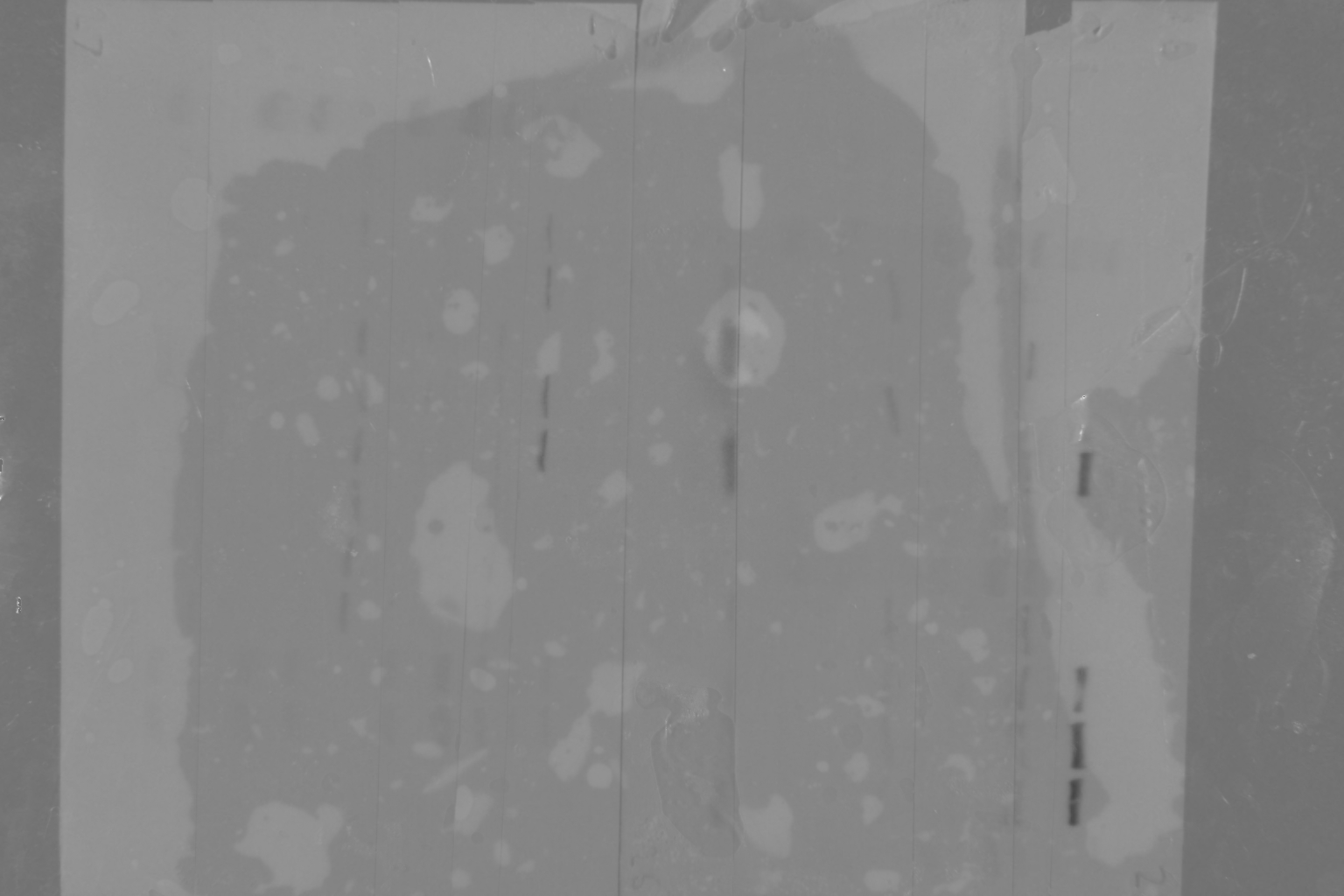

Supplement: Figure 5—source data 2. [file elife-98072-fig5-data2.zip › 20230105_1102_26_27_1_4sec.tif_overlay.tif]
